# Epigenetic Therapies in Triple-Negative Breast Cancer: Concepts, Visions, and Challenges

**DOI:** 10.3390/cancers16122164

**Published:** 2024-06-07

**Authors:** Ulrich Lehmann

**Affiliations:** Institute of Pathology, Hannover Medical School, Carl-Neuberg-Str. 1, D-30625 Hannover, Germany; lehmann.ulrich@mh-hannover.de; Tel.: +49-(0)511-532-4501; Fax: +49-(0)511-532-5799

**Keywords:** triple-negative breast cancer, epigenetics, DNA methylation, histone modification, targeted therapy

## Abstract

**Simple Summary:**

Triple-negative breast cancer is an aggressive subtype of this frequent malignancy in women, for which new therapeutic options are urgently needed. Changes in the packaging of the chromosomes within the cell nucleus, so called “epigenetic modifications”, cooperate with direct changes of the DNA sequence, called “genetic alterations”, in the development and progression of human tumors. The definition of epigenetic phenomena and the identification of the underlying molecular mechanisms is an active field of research with many open questions. So far, the best-defined players in the field of epigenetics are the methylation of DNA and the covalent modification of histone proteins around which the DNA is wrapped in the nucleus of human cells. Both modifications affect the expression of genes. The principal reversibility of these epigenetic modifications of the DNA strands and the histone proteins make them an attractive target for therapeutic intervention by so-called “epidrugs”, and several promising clinical trials are under way. However, many questions regarding specificity, safety, and efficiency of these drugs are still unresolved.

**Abstract:**

Breast cancer, the most frequent malignancy in women worldwide, is a molecularly and clinically very heterogeneous disease. Triple-negative breast cancer is defined by the absence of hormone receptor and growth factor receptor ERBB2/HER2 expression. It is characterized by a more aggressive course of disease and a shortage of effective therapeutic approaches. Hallmarks of cancer cells are not only genetic alterations, but also epigenetic aberrations. The most studied and best understood alterations are methylation of the DNA base cytosine and the covalent modification of histone proteins. The reversibility of these covalent modifications make them attractive targets for therapeutic intervention, as documented in numerous ongoing clinical trials. Epidrugs, targeting DNA methylation and histone modifications, might offer attractive new options in treating triple-negative breast cancer. Currently, the most promising options are combination therapies in which the epidrug increases the efficiency of immuncheckpoint inhibitors. This review focusses exclusively on DNA methylation and histone modifications. In reviewing the knowledge about epigenetic therapies in breast cancer, and especially triple-negative breast cancer, the focus is on explaining concepts and raising awareness of what is not yet known and what has to be clarified in the future.

## 1. Introduction: Epigenetics—Concepts, Misconceptions, and a Working Definition

Epigenetics is one of the most contested and enigmatic concepts in modern biology [1,2], with a long history dating back far beyond Conrad Waddington’s seminal work 80 years ago [3]. In medicine, “epigenetics” is overcharged with hopes and enthusiasm for resolving still-not-understood phenomena and for addressing unmet needs in the therapy of all kinds of diseases [4]. Unfortunately, in stark contrast to its popularity (or perhaps the very reason for this popularity?), the meaning of “epigenetics” is very often far from clear, and countless definitions can be found in the literature (e.g., Table 1 in [5]). At least two strands of reasoning can be identified: changing expression patterns during development (a phenomenon referred to by Waddington [3]), or mechanisms of inheritance somehow “beyond” the primary DNA sequence and which are not detectable by conventional DNA sequencing [6,7].

Quite a few researchers envision “epigenetics” as a confirmation of Lamarck’s theory on the inheritance of acquired traits. However, on closer inspection, there are many indications of serious misconceptions about what Lamarck really said and had in mind. The correct delineation of this line of reasoning, and the adequate discussion of all positions in this field, is well beyond the scope of this review. Therefore, the interested reader should consult the following reviews and the references therein: [4,8].

Many observations ascribed to epigenetic phenomena or mechanisms are actually consequences of intrauterine exposure, and have nothing to do with transgenerational inheritance [9,10] of collected and somehow or somewhere stored “life experiences”. Unfortunately, the line between serious science and carefully controlled thought-provoking observations on the one hand, and pseudoscience and dubious anecdotes on the other hand, is often crossed in this otherwise interesting field.

Another challenge in discussing “epigenetics” is the fact that mechanisms of inheritance in plants and fungi are, in several aspects, fundamentally different from those in animals (see: [11,12,13] and the references therein). There are many interesting epigenetic phenomena and mechanisms in fungi or plants, but as these have no medical relevance for humans, and so they are not discussed here. This review focusses exclusively on the question of whether, and how, alterations in DNA methylation patterns and covalent histone modifications could be used to improve the therapy of triple-negative breast cancer. This deliberately leaves out many biological phenomena and molecular mechanisms often also subsumed and discussed under the headline “epigenetics” or “epigenetic therapy”, such as, most prominently, all sorts of non-coding RNA species ([14] and the references therein) and RNA modifications ([15] and the references therein).

## 2. Epigenetic Phenomena

In order to develop meaningful new approaches in the field of epigenetic therapy, one has to distinguish between epigenetic phenomena and the underlying molecular mechanisms. Only the latter mechanisms can be targeted by epidrugs. Table 1 provides an overview about the best-known epigenetic phenomena.

## 3. Epigenetic Mechanisms

Various molecular mechanisms are responsible for the epigenetic phenomena listed in Table 1: DNA methylation, histone modifications, microRNA, chromatin-remodeling complexes, polycomb/trithorax complexes, long ncRNA, RNA modifications, and various small ncRNAs, among others. The interested reader is referred to the following reviews and the references therein: [22,23,24]. For the following reasons, this review will concentrate exclusively on DNA methylation (at the DNA base cytosine) and covalent histone modifications (with a focus on histone acetylation and methylation):

- They are best studied and understood in molecular terms.
- The analytical tools to identify these modifications and monitor alterations during the course of disease or after therapeutic intervention are well developed.
- Aberrations in DNA methylation and histone modifications are well described in many human diseases, especially in human cancers.
- Targeting DNA methylation and/or histone modifications has been studied in many clinical trials in oncology.
- Drugs targeting DNA methylation and histone modifications are approved and are available for treating human cancer patients.

A comprehensive overview about all the described and functionally relevant histone modifications can be found in the following reviews: [25,26].

### 3.1. DNA Methylation

In humans, DNA methylation takes place at the carbon atom number 5 of the pyrimidine ring of the DNA base cytosine if this cytosine is followed by a guanosine. Therefore, it is often called “CG methylation” or “CpG methylation” (“p” stands for the phosphodiester backbone of the DNA strand, [27]). The dinucleotide CG is much less frequent than the other theoretically possible and naturally occurring dinucleotides (AA, AT, AC, and so forth), a phenomenon called “CG suppression” [28]. There are small regions in the human genome that are rich in CG dinucleotides, the so-called “CpG islands”, half of which are associated with the 5′ end of genes [29]. DNA methylation is tightly linked to transcriptional repression [30]. In most cases, however, DNA methylation does not lead to direct repression of gene expression, but rather seems to enhance transcriptional repression through regulatory events [23,24]. DNA methylation within a gene, so-called “gene body methylation”, can even stimulate transcription [31].

The fact that DNA methylation has a role “in the generation of tumor heterogeneity and progression” was proposed nearly 40 years ago [32], and was confirmed in countless experiments and numerous publications in the following decades [33,34,35]. The retinoblastoma gene *RB1*, encoding the cell-cycle regulator pRb [36], was the very first *bona fide* tumor suppressor gene shown to be inactivated by aberrant DNA methylation [37]. This epigenetic inactivation can be considered to be a “functional deletion”, which complements inactivation by genetic mechanisms. It can occur even much more frequently than the textbook mechanism of tumor-suppressor gene inactivation by deleterious genetic alterations [38].

### 3.2. Histone Modification

“Histone modification” refers to the covalent modification of the unstructured N-termini of the histone core proteins, mostly at lysine, arginine, and serine residues [25]. It critically modulates the protein–protein and protein–DNA interactions, thereby affecting the regulation of gene expression. For a long time, these histone tails were totally neglected in chromatin research, because, in order to obtain high-resolution crystal structures, these unstructured tails had to be removed. Therefore, many textbook illustrations contain these N-termini only as dotted lines or not at all (see [39] and the references therein). Under special circumstances, the prototypical histone proteins can be exchanged against so-called “variant histone proteins” with different biological properties [40]. The biological activity of histone proteins can also be affected by mutations, especially at the potential modification sites in the N-terminus (e.g., codon 27 in histone H3). This occurs quite frequently in several brain tumors [41].

**Figure 1:**
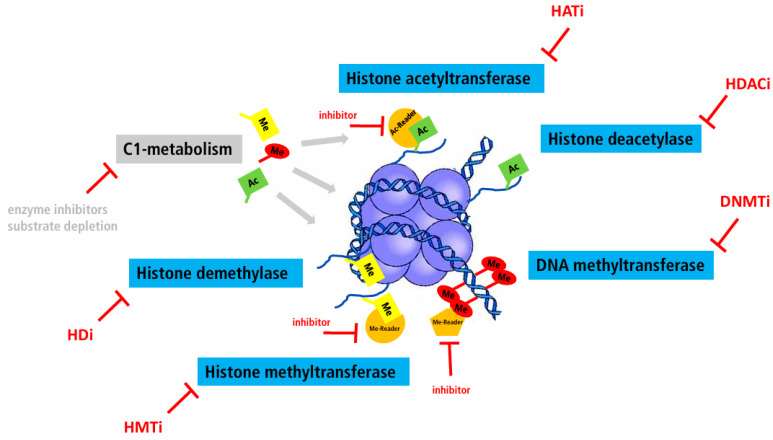
Overview of possible “directed epigenetic therapy” approaches for the treatment of cancer. Ac: Acetyl group, DNMTi: DNA methyltransferase inhibitor, HATi: Histone acetyltransferase inhibitor, HDi: Histone demethylase inhibitor, HMTi: Histone methyltransferase inhibitor, Me: Methyl group.

The insightful review by Zhao et al. [42] defines not only the well-known “writers” (HAT, HMT, DNMT, and others), “readers” (e.g., bromodomain or methyl binding domain), and “erasers” (HDAC, HDM, and others), but also the “paper” (histone variants, chromatin remodelers, etc.), the “ink” (methyl- or acetyl-group donors, etc.), and the “bookbinding” (chromatin looping, chromatin phase separation, etc.). Figure 1 in reference [42] provides a comprehensive overview of these concepts and the terminology. Principally, each of these components represents a target for pharmacological intervention (inhibition, activation, depletion, replenishment, etc.), but so far, most functional studies and clinical trials focus only on a small group of writers and erasers as promising therapeutic targets.

## 4. Epigenetic Therapy

### Definition

The fact that most chromatin factors are enzymes (see Figure 1) make them prototypical targets for specific inhibitors, especially when they are overexpressed. In this review, “epigenetic therapy” refers to the modulation of DNA methylation and histone protein modification, aiming at the normalization of DNA methylation and histone modification patterns.

This can be accomplished by small molecules (sometimes called “epidrugs”) or site-specific genetic engineering:

- Inhibition of DNA methyltransferases (DNMTi).
- Overexpression or activation of DNA methyltransferases (DNMT).
- Inhibition of histone-modifying enzymes (e.g., histone deacetylase inhibitors, HDACi).
- Overexpression or activation of histone-modifying enzymes.
- Inhibition or activation of DNA methyltransferases or histone-modifying enzymes by substrate depletion.
- Targeted removal or addition of methyl groups.
- Targeted modification of histone proteins.

Despite the fact that enzymes are attractive targets whose activity can be modulated by various means, the principal problem of this approach is the lack of specificity: all cell types are targeted, and within one cell, countless genomic loci are targeted. Under many circumstances, this might represent a principal barrier for the development of useful drugs based on this approach, and will require extensive empirical testing for each new drug in clinical trials, which in itself represents a formidable challenge. In addition, this lack of specificity might explain the lack of useful validated predictive markers in the clinics, indicating favorable response to the epidrug.

Principally, the most specific “epigenetic” approach would be the gene-specific removal or addition of methyl groups, thereby activating or repressing, in the best scenario, the expression of a single transcript.

So far, the vast majority of clinical trials employing one or the other epidrug rely on the inhibition of an enzyme or a whole class of enzymes without any gene specificity and only a limited cell-type specificity.

During cell division, the whole chromatin (chromosomes plus histone proteins) has to be duplicated. This requires increased activity of DNA methyltransferases and all sorts of histone-modifying enzymes in order to faithfully replicate all DNA and histone modifications. To achieve this in time, these enzymes are upregulated during cell division. For these reasons, epigenetic therapies employing enzyme inhibitors always affect healthy, replicating cells, like conventional chemotherapy. This explains also why many enzymes involved in DNA and histone modifications are upregulated in tumor cells, and why many reports about specific activation of the encoding genes in tumor cells are false positive findings which disappear if proper controls for proliferation-associated effects are employed (already shown more than 20 years ago, e.g., [43]).

For the complex relationship between chromatin structure (i.e., DNA methylation and histone modifications) and the cellular metabolism, see the excellent and comprehensive review by Li et al. [44].

## 5. Epigenetics Therapy—The Beginnings

### Aza-Cytidine

DNA methyltransferase inhibitors, and other agents interfering with DNA methylation, are collectively called “hypomethylating agents, (HMA)”. Initial experiences using nucleotide analogues as DNMT inhibitors in treating cancer patients were not very encouraging. The cytotoxicity of HMA often outweighs any potential therapeutic benefit due to demethylation (see [45] and the references therein).

A major breakthrough in the use of epigenetic drugs for the treatment of human malignancies was the development of slow low-dose application schemas, which showed much better response rates and less toxicity [46,47,48]. This therapeutic approach is especially useful for patients with Myelodysplastic Syndrome (MDS) and in elderly, unfit AML patients not eligible for standard induction chemotherapy [49].

## 6. Single Agent versus Combination Therapy

In many clinical situations, a combination of drugs is more efficient, increasing potency while reducing toxicity and delaying the development of resistance.

However, modulation of DNA methylation might interfere with effectiveness of chemotherapy. Aberrant DNA methylation and the accompanying silencing of the *MGMT* or *BRCA1* gene renders cells more sensitive against alkylating or DNA-damage-inducing compounds. Pharmacological demethylation would therefore interfere with the effectiveness of chemotherapy. By contrast, the inactivation of genes involved in engaging cell death responses, like *MLH1*, is associated with chemoresistance. Targeted demethylation would increase response to chemotherapy in this scenario. Therefore, the selection of combination therapy regimens depends on the DNA methylation profile of the target cells [50].

The treatment of patients with demethylating agents will always lack specificity. Therefore, extensive empirical testing will be required (in carefully designed clinical trials), and prediction of responses will remain challenging.

Molecularly targeted approaches employing recently developed tools for precise site-specific editing of the human genome are promising (see below). However, they are time consuming and very costly. Therefore, it remains to be seen whether these tools are a viable option in the clinical setting with a very often urgent need for initiating therapy.

## 7. Breast Cancer

Breast cancer is the most frequent malignancy in women, with more than 2.26 million new cases worldwide in 2020. In total, 339,350 new cases in 2022 in the US alone, and 374,800 new cases in the European Union in 2022 (https://ecis.jrc.ec.europa.eu/pdf/Breast_cancer_2022-Oct_2023.pdf; accessed on 27 November 2023). In contrast to the public perception, “breast cancer” is not a single disease, but a very heterogeneous group of diseases with a huge variability in the course of disease (measured as a percentage of cured patients, duration of progression-free survival, and overall survival). In addition, the treatment options vary substantially according to the molecular characteristics of the different subtypes [51,52].

Array-based mRNA expression profiling has revolutionized the traditional morphology-based classification of breast cancer specimens [53,54,55]. The attempts to transfer these mRNA-profiling-based molecular categorizations into an immunohistochemical test that can be performed more broadly in the routine diagnostic setting are described and critically discussed in Scymiczek et al. [56]. The breast cancer subgroup negative for hormone receptor and ERBB2/Her2 growth factor receptor expression (ER, PR, and ERBB2/HER2-negative) was named “triple-negative (TN)” breast cancer, and was initially thought to be more-or-less identical with the molecularly defined “basal” subtype. Subsequent studies revealed that this breast cancer subtype is in itself heterogeneous and comprises several TN-subtypes (see [57] and the references therein). Clinically, in most breast cancer cases, triple negativity is associated with unfavorable prognosis and limited therapeutic options, and, consequently, with many failed clinical trials [58]. Therefore, new concepts and drugs are urgently needed for the treatment of triple-negative breast cancer.

Of note, the recent and very comprehensive review from Bianchini G. et al., full of insights about the “treatment landscape of triple negative breast cancer”, with over 200 references [59], does not elaborate on the potential integration of epigenetic drugs into the treatment of triple-negative breast cancer. “DNA methylation” is mentioned by Bianchini et al. only in the context of *BRCA1* gene methylation. The *BRCA1* gene methylation represents an alternative mechanism for inactivation, functionally equivalent to deleterious genetic mutations, as an underlying cause for Homologous Recombination Deficiency (HRD), which is a therapeutic target for PARP inhibitors [60].

Glodzik et al. ([38], not cited by Bianchini et al. [59]) show, in a Swedish cohort of triple-negative breast cancer, that *BRCA1* inactivation due to promotor hypermethylation is twice as frequent in triple-negative breast cancer as *BRCA1* inactivation due to a pathogenic mutation, and that it “confers an HRD, immune cell type, genome-wide DNA methylation, and transcriptional phenotype similar to TNBC tumors with *BRCA1*-inactivating variants”. This offers the opportunity for *BRCA1* reactivation in a substantial number of triple-negative breast cancer cases. However, in this scenario, one has to consider that loss of BRCA1 repair activity in itself offers a therapeutic target by increasing the sensitivity of the affected cells against platin-containing chemotherapeutics and PARP inhibitor therapy. Therefore, the benefits of both approaches have to be weighed against each other after considering all clinical parameters.

The advantage of this reactivation approach is that it is much easier (and independent of the patient-specific sequence context) to reinduce the expression of a silenced gene than to re-engineer a gene with a pathogenic variant which causes nonsense-mediated degradation of the encoded protein. The functional restoration of epigenetically inactivated genes by administration of mimetic drugs is explained in great detail by Dahl et al. [61].

Currently, the trial landscape for epidrugs in triple-negative breast cancer is quite heterogeneous. Due to subtle differences in the trial design, it is often difficult to compare different trials, and some trials enrolled only a few patients, complicating the generalizability of the findings.

## 8. Epigenetic Subtypes in Human Breast Cancer

Shortly after transcription profiling identified the intrinsic molecular subtypes of human breast cancer DNA, methylation profiling was initiated in order to identify epigenetic subtypes. Starting from small-scale profiling approaches, most recent publications describe the more-or-less genome-wide characterization of DNA methylation patterns in patient samples. The following references provide an overview of the methodological approaches: [62,63,64,65,66,67,68]. Because a wide range of various methods has been used for the analysis of different study cohorts, direct comparison of results can be challenging.

The EZH2 inhibitor Tazemetostat (Tazverik™) was the first inhibitor of a histone “writer” approved for treatment of a solid tumor [69]. However, activating mutations in the *EZH2* gene are very rare in solid tumors, especially in human breast cancer, and, therefore, only a subgroup of breast cancer patients might profit from this approach (approx. 0.1% in human breast cancer, cBioPortal).

Also, mutations in other “epigenetic genes”, which are targets for approved drugs in AML and cholangiocellular carcinoma [70], are quite rare in breast cancer. A cBioPortal search in 6344 invasive breast cancer specimens revealed, for example, only four cases with *IDH1* hotspot mutations (all p.R132C), and no cases whatsoever with an *IDH2* hotspot mutation. Whether the copy number changes found in a subgroup of human breast cancer specimens (50 and 100 cases for *IDH1* and *IDH2*, respectively) have any functional and clinical relevance in this context is currently not known.

Stirzaker C. et al. [71] were able to identify three distinct methylation patterns in triple-negative breast cancer samples (histological grade 3) by employing MDB-affinity capture-based sequencing (MDBCapSeq). These DNA methylation clusters are associated with the prognosis of the TNBC patients. Future studies have to show whether these clusters are also associated with different response to epigenetic drugs, especially hypomethylating agents. Lin L.H. et al. [72] also independently identified three epigenetic subtypes of triple-negative breast cancer. However, this was identified without any prognostic association, indicating that additional research addressing the prognostic value of DNA methylation subtypes in triple-negative breast cancer is necessary.

Zolota V. et al. highlight, in their recent review about epigenetic alterations in triple-negative breast cancer [73], the importance of the interaction between tumor cells and the extracellular matrix, and that nearly all components of this complex interplay can be affected by epigenetic aberrations. This identifies additional opportunities for therapeutic intervention (DNA methylation and histone-modification inhibitors, as well as drugs targeting other epigenetic mechanisms affecting the tumor cell–extracellular matrix interaction). However, this still requires, according to the authors, more and better clinical trials before firm conclusions can be drawn.

## 9. “Directed Epigenetic Therapy” in Triple Negative Breast Cancer

“Directed epigenetic therapy” comprises all approaches targeting more-or-less specifically one component of the epigenetic machinery, e.g., using DNA methytransferase inhibitors or histone acetylase inhibitors. As explained above, these enzyme-directed approaches suffer from an inherent lack of specificity, and are reviewed extensively elsewhere ([74,75,76] and the references therein). Table 2 lists a selection of clinical trials employing this approach.

## 10. Epigenetic Therapy as “Conditioner”

Epigenetic therapy as “conditioner” comprises all approaches which try to modify the epigenome in order to enhance the efficiency of another drug (or drug combination). An example is the reactivation of transposable elements in the human genome by demethylating agents, which could increase the number of neoantigens expressed on the cell surface, which in turn could increase the efficiency of immune checkpoint inhibitors. In addition, the expression of cancer testis antigens is reported to be reactivated using DNA methyltransferases [77]. Ten years ago, it was already demonstrated that pharmacological modulation of the DNA methylation landscape can potentiate the effectiveness of immunotherapy approaches [78,79]. Subsequent studies explored the use of hypomethylating agents (HMA) to induce viral mimicry and increase tumor immunogenicity (see [80] and the references therein). Pioneering studies also showed that the inhibition of histone-modifying enzymes can increase the effectiveness of an immune checkpoint inhibitor therapy [81].

In principal, epidrugs can make cancer cells more susceptible and/or immune cells more effective. The former effect is based on the reactivation of endogenous retroviral (ERV) sequences within the tumor cell genome, which activates interferone-based anti-tumor cell activities [78,79]. The latter mechanism is convincingly demonstrated by Loo Yau H et al., who showed increase in activation and cytolytic activity of CD8+ T cells after administration of HMA [82].

Micevic G. et al. [83] provide a recent overview about this type of combination therapy for various solid tumors. Table 3 lists the trials that focus on triple-negative breast cancer.

Birabresib is the only drug listed in Table 2 which targets a “reader” (i.e., a protein binding to specific histone modifications, see Figure 1). To et al. [84] provide a comprehensive review about this class of epidrugs and recent developments to overcome still-existing limitations (mostly related to the lack of cell and gene specificity). A promising approach seems to be the combination of BET inhibition with conventional chemotherapy and an immune checkpoint inhibitor [85].

The interested reader should routinely check every study on clinicaltrials.gov, which is continuously updated and represents the most comprehensive description of the trial design, the timeline, and the actual recruitment. In preparing the current manuscript, it was revelead that several compilations in clinical trials employing epidrugs contain inconsistencies due to typing errors or missed updates (e.g., Table 1 in [86]: NCT03295552 is terminated; NCT04296942 is terminated due to new safety data; NCT02957968 is active, and recruitment does not fit to the title of the table and the title of the review).

A major hurdle for the successful application of epidrugs in the treatment of triple-negative breast cancer is the still not completely resolved heterogeneity of this subtype (which actually comprises several “subtype-subtypes”, see: [57]) and the lack of validated clinically useful biomarkers for predicting treatment response. The field of hematology is much further developed regarding this question of predictive biomarkers for epigenetic therapy [87].

Meyer B et al. [88] presented promising data about differential DNA methylation in triple-negative breast cancer as a predictive marker for responses to chemotherapy. However, predictive markers for epigenetic therapy are not yet identified.

## 11. Epigenetic Therapy by Genome Editing

A very interesting and promising approach in the field of epigenetic therapy is the use of CRISPR-Cas9-based genome editing protocols (for a very recent comprehensive review, see: [89]) for the reversal of DNA methylation and histone modification aberrations in cancer cells [90,91]. This concept would introduce the required specificity into the field of epigenetic therapy by precisely defining which genomic region is modified via the Cas9 guide RNA sequence. This will enable the demethylation of a single promotor affected by aberrant hypermethylation in cancer cells and the subsequent reactivation of the expression of this gene. Similarly, histone modifications could be removed at a single promotor without affecting any other gene in the cancer cells.

A principal challenge will be the fact that, under most circumstances, epigenetic aberrations, in the form of altered DNA methylation or histone modification patterns, lead to a very dense chromatin structure at the affected loci. This might pose major hurdles for the accessibility of the regions of interest (for details and further references, see: [91]). Another challenge will be the turn-around time in which all required tools for the epigenenome editing can be prepared and applied. In a wider perspective on the healthcare system, questions of costs, reimbursement, and ethical considerations also have to be kept in mind if genome editing is applied in treating human beings [92,93].

## 12. Conclusions and Future Perspectives

After a long and often frustrating “incubation period”, the field of epigenetic therapy of solid tumors has seen a tremendous increase in the molecular understanding of the pharmacological modulation of epigenetic aberrations in human tumor cells, thereby identifying new promising targets and possibilities for combination therapies.

The systematic exploitation of 3D organoid cultures for drug screening (see [94] for a recent example) will clearly facilitate and accelerate the transfer of new compounds from the laboratory into the clinics, which are still a major bottleneck for epigenetic drugs. The accumulated evidence from previous decades underscore the importance of the pharmaceutical form of the epigenetic drug, and make combination therapies the most promising avenue for progress in this rapidly developing field.

This gain in theoretical and practical knowledge provides some hope for an improvement in the treatment of triple-negative breast cancer in the foreseeable future.

## Figures and Tables

**Table 1:** Selected epigenetic phenomena.

	Reference
Phenotypical variability of identical twins	[16]
X-chromosome inactivation	[17]
Mating types in *S. cerevisiae*	[18]
Imprinting	[19]
Functional and phenotypical variability of cells in higher organisms	[20]
Epiphenotypes in *Arabidopsis* flowers	[21]
Phenotypical variability of cloned animals	[20]

**Table 2:** Selected trials of epigenetic drugs in triple-negative breast cancer.

	Reference
Birabresib (BETi)	NCT02259114
Entinostat (HDACi) + Azacytidin (DNMTi)	NCT01349959
Decitabine (DNMTi) + Carboplatin	NCT03295552
Chidamide (HDACi) + Cisplatin	NCT04192903
Entinostat (HADCi) + other drugs	NCT04296902
Abbreviations: BETi: bromodomain and extra-terminal motif inhibitor, DNMTi: DNA methylatransferase inhibitor, HDACi: Histone deacetylase inhibitor	

**Table 3:** Selected trials in triple-negative breast cancer combining epigenetic drugs and immune checkpoint inhibitors.

	Reference
Panobinostat (HDACi)/Everolimus/LCL161 + PRD001 (anti-PD-1)	NCT02890069
Entinostat (HDACi) + Atezolizumab (anti-PD-L1)	NCT02708680
RO6870810 (BETi) + Atezolizumab (anti-PD-L1)	NCT03292172
Abbreviations: BETi: bromodomain and extra-terminal motif inhibitor, HDACi: Histone deacetylase inhibitor	
